# Comparative Genomics of a Polyvalent *Escherichia-Salmonella* Phage fp01 and In Silico Analysis of Its Receptor Binding Protein and Conserved Enterobacteriaceae Phage Receptor

**DOI:** 10.3390/v15020379

**Published:** 2023-01-28

**Authors:** Ignacio Vasquez, Julio Retamales, Barbara Parra, Vimbai Machimbirike, James Robeson, Javier Santander

**Affiliations:** 1 Marine Microbial Pathogenesis and Vaccinology Laboratory, Department of Ocean Science, Memorial University, St. John’s, NL A1C 5S7, Canada; 2 Instituto de Ciencias Naturales, Universidad de las Américas, Viña del Mar 2520000, Chile; 3 Subdepartment of Molecular Genetics, Public Health Institute of Chile, Santiago 9140000, Chile; 4 Laboratory of Microbiology, Institute of Biology, Pontifical Catholic University of Valparaíso, Valparaiso 2370000, Chile

**Keywords:** polyvalent bacteriophage fp01, *Escherichia coli*, *Salmonella*, genome

## Abstract

The polyvalent bacteriophage fp01, isolated from wastewater in Valparaiso, Chile, was described to have lytic activity across bacterial species, including *Escherichia coli* and *Salmonella* enterica serovars. Due to its polyvalent nature, the bacteriophage fp01 has potential applications in the biomedical, food and agricultural industries. Also, fundamental aspects of polyvalent bacteriophage biology are unknown. In this study, we sequenced and described the complete genome of the polyvalent phage fp01 (MH745368.2) using long- (MinION, Nanopore) and short-reads (MiSeq, Illumina) sequencing. The bacteriophage fp01 genome has 109,515 bp, double-stranded DNA with an average G+C content of 39%, and 158 coding sequences (CDSs). Phage fp01 has genes with high similarity to *Escherichia coli*, *Salmonella enterica*, and *Shigella* sp. phages. Phylogenetic analyses indicated that the phage fp01 is a new *Tequintavirus* fp01 specie. Receptor binding protein gp108 was identified as potentially responsible for fp01 polyvalent characteristics, which binds to conserved amino acid regions of the FhuA receptor of Enterobacteriaceae.

## 1. Introduction

Bacteriophages or phages are bacterial viruses characterized by their obligatory bacterial parasitism, influencing bacterial ecology and evolution [1,2]. Since the early 1900s, lytic bacteriophages have been utilized as prophylactic and therapeutic agents against bacterial infectious diseases [3,4]. A large amount of research proves the effectiveness and safety of bacteriophages utilization [5,6]. Nowadays, the utilization of bacteriophages has public acceptance and government approval [7]. Commercial bacteriophage cocktails are currently utilized in human and animal health, and in the Agri-food industry to prevent bacterial infectious diseases [4,8]. Bacteriophage host-range is typically narrow, and lytic bacteriophages are usually species-specific or even strain-specific [9]. Most of the bacteriophages possess a tail that allows specific recognition and subsequent adsorption to a receptor at the surface of the host bacterium [10,11]. Because of phage-host specificity, phage cocktails or mixes that offer a broad host-range are frequently utilized in commercial preparations [12,13].

Polyvalent phages have been described since 1933 [14], including phages of Enterobacteria [11] and staphylococci [15], *Aerobacter aerogenes* [16], and *Pseudomonas* spp. [17]. Polyvalent phages that can infect different bacteria species or serotypes are very attractive for industrial applications. Polyvalent phages offer the possibility of increasing bacterial species coverage of the phage cocktails [12,13], and to propagate the bacteriophages in non-pathogenic hosts, reducing the risk of accidental contamination of preparations with the target pathogen.

The polyvalent bacteriophage fp01 was isolated from wastewater in the V region of Chile, using *Salmonella enterica* serotype Choleraesuis VAL201 as the host [18]. Previous taxonomic analyses of the phage fp01 indicated that this phage belongs to the order *Caudoviridae*, family *Siphoviridae,* which are bacterial viruses of double-strand DNA (ddDNA) [18]. However, current taxonomy described this bacteriophage as a member of the class *Caudoviricetes*, family Demerecviridae, with a siphovirus morphology [19]. The bacteriophage fp01 is able to proliferate in *E. coli* C, *E. coli* B, *E. coli* K12, and *Salmonella enterica* serovars Typhi, Paratyphi B, and Choleraesuis [18], indicating that fp01 has a common attachment site on the susceptible bacterial species [18,20].

The interaction between the phage and the Receptor-Binding-Proteins (RBPs) results in the release of the phage DNA into the bacterial host. Previous studies demonstrated that phage interaction with the RBPs at the bacterial outer membrane increases phage propagation [21,22,23]. For example, the interaction between the T5 phage fimbria protein pb5 and the *E. coli* RBP, ferrichrome transport FhuA, triggers the release of the phage DNA. This interaction is mediated by the *β*-barrel structure and external loops of FhuA [21,22,23].

A better understanding of the mechanisms and evolution of polyvalent bacteriophages can be obtained by comparative genomic analysis. Here, we sequenced and described the whole genome of the polyvalent bacteriophage fp01 using long-reads (MinION, Nanopore) and short-reads (MiSeq, Illumina, San Diego, CA, USA) technologies. We found that the phage fp01 possesses a complete replication machinery but depends on host factors for transcription. Several genes associated with recombination and DNA cleavage, as well as cell lysis components, were identified in the genome of fp01. Phylogenetic analyses indicated that the phage fp01 is closely related to the *Tequintavirus* genus, which contains phages such as *Escherichia* phage T5_ev219 and *Salmonella* virus VSe12. The Phage Binding Protein (PBP) gp108 was identified in fp01 as pb5-like PBP (YP_009841487.1), which potentially could interact with conserved binding residues THR553, THR555 and ASN 556 present in FhuA enterobacterial receptor.

## 2. Materials and Methods

### 2.1. DNA Extraction

The bacteriophage fp01 was propagated in *S. Choleraesuis* VAL201 using standardized methods [24,25]. Genomic DNA (gDNA) from concentrated phage lysates was purified according to the method described by Kaiser et al. [26]. DNA was quantified and tested for purity (260/280 ratio) using spectrophotometry in a Genova-Nano spectrophotometer (Jenway, Staffordshire, UK).

### 2.2. Sequencing and Genome Assembly

The MinION is a USB-portable and low-cost device, which can generate reads of 2–10 Kb on average, with an error range of 2–13% [27,28,29,30], ideal for sequencing small genomes like the bacteriophage fp01. The MinION sequencing library was prepared using the SQK-RAD003 kit according to the manufacturer’s instructions and sequenced using an R9.Spot-On flow cell (FLO-MIN106) (Oxford Nanopore, NY, 10013, USA). The fp01 gDNA library was added to a MinION sequencer and run for 22 h with coverage of 29.68×. Coverage was calculated by the Lander-Waterman equation [31]. The resulting FAST5 files were based-called and demultiplexed using Albacore v2.0.2. The FAST5 files were converted into FASTA format using Poretools [8]. The contigs were analyzed and visualized using CLC Genomics Workbench 20 (CLCBio, Qiagen, Aarhus, Demark).

Additionally, libraries and sequencing were conducted commercially at Genome Quebec (Canada) and sequenced using the Miseq Illumina platform. The quality of reads was evaluated using FastQC v.12 (Babraham Institute, Cambridge, UK) [32]. Illumina Mi-Seq sequences were trimmed and assembled using CLC Genomics Workbench (CLCBio) v.20.0 (Qiagen, Demark) de novo and genome finishing module tools with default parameters.

### 2.3. Annotation and Genome Mapping

The genome was initially annotated with the PHAge Search Tool (PHASTER; https://phaster.ca/) [33] (accessed on 18 January 2023) and refined with the Rapid Annotation Subsystem Technology (RAST; https://rast.nmpdr.org/) [34] (accessed on 18 January 2023). The whole genome was submitted to NCBI data using the whole genome shotgun submission pipeline (WGS) (https://www.ncbi.nlm.nih.gov/genbank/wgs/ accessed on 1 December 2022). The phage fp01 genome was deposited in DDBJ/EMBL/GenBank under the BioProject (PRJNA450422) and the accession number (NC_048731.1). The phage fp01 genome was mapped and visualized using the CGView server (http://cgview.ca/, accessed on 18 January 2023).

### 2.4. Comparative Genomics and Phylogenetic Analysis

Forty-one bacteriophage genome sequences listed in Table 1 were aligned using the CLC whole genome analysis tool by default parameters (Min. initial seed length = 15; Allow mismatches = yes; Min. alignment block = 100; Min. similarity (0.8); Min. length (0.8)). Average nucleotide identity (ANI) and alignment percentage (AP) were calculated based on the aligned genomes. A heat map was computed based on the previous alignment using the heat-map tool with default parameters (Euclidean distance method and complete cluster linkages). Closely related bacteriophage genomes were selected for further comparative synteny analysis. Dot plots were generated to represent homologous regions, orthologs, genome gaps (GGs), and inversions within the genomes. The evolutionary analyses of the whole genome of the phage fp01 were conducted using MEGAX. The Neighbor-Joining method [35] with a bootstrap test of 1000 replicates and the Jukes–cantor method [36] was utilized to determine the evolutionary distances. The Enterobacteria bacteriophage M13 genome was used as out group for the analysis.

### 2.5. Protein Modelling and Molecular Docking

FhuA (ferric receptor/phages binding receptor) protein sequence from *Escherichia coli* K-12 (NC_000913.3), *Salmonella enterica* serv. Choleraesuis ATCC 10708 (AKW03981.2), *Salmonella enterica* serv. Paratyphi B CFSAN016062 (EDC2010892.1), *Salmonella enterica* serv. Typhi 1242879 (EHS1467780.1) were obtained from NCBI and aligned in ESPript3 [50], using *E. coli* K-12 FhuA PDB_1FCP structure [22] as reference. FhuA 3D structure view was obtained from RCSB Protein Data Bank.

The phage binding protein (PBP) of bacteriophage fp01, was identified by BlastP search in the NCBI database against the PBP tail protein Pb5 (AAX12083.1) from *E. coli* phage T5. The Pb5 protein was shown to bind with the FhuA protein [23], but Pb5 structural protein confirmation is not yet available. Therefore, due to the absence of a protein template for Pb5 protein, the tertiary structure of gp108 was modeled ab initio using trRosetta webserver [51] refined by GalaxyRefine implemented in GalaxyWeb webserver (https://galaxy.seoklab.org/) [52]. The quality of the protein model was analyzed using the SWISS-MODEL webserver (https://swissmodel.expasy.org/) [53]. Consequently, the FhuA receptor (PDB ID: 1FCP) [22] was modeled as a transmembrane protein using the PPM 2.0 webserver (https://opm.phar.umich.edu/ppm_server2) [54]. Protein-protein molecular docking was performed by the HDOCK webserver (http://hdock.phys.hust.edu.cn/) [55] using gp108 as the ligand and FhuA as the receptor. The predicted binding affinity and dissociation constant were calculated using the PRODIGY webserver (https://wenmr.science.uu.nl/prodigy/) [56]. Analyses for gp108 modeling were computed using the software’s default parameters. The last accessed date for all the webservers described was 18 January 2023.

## 3. Results

### 3.1. Sequencing

**Figure 1:**
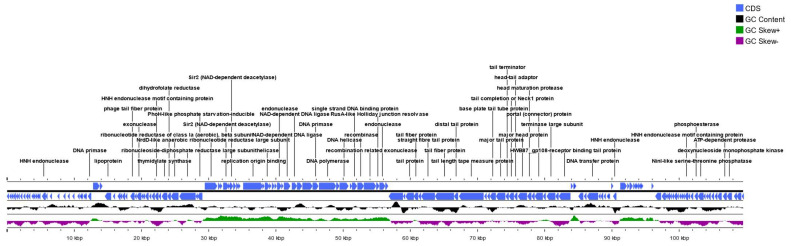
*Escherichia* phage fp01 genome map. Genome map visualization of polyvalent bacteriophage fp01. Mapping was performed using the CGViewer Server pipeline. Color arrows represent CDS (blue); GC content (black), GC Skew (+) (forest green), and GC Skew (−1) (violet).

Using the long-read sequencing technology, the total analyzed bacteriophage fp01 reads were 2067 with 7546 nt on average (Figure S1A). Only 12 reads did not pass the quality control and were removed from the analysis (Figure S1B,C). The percentage of successful sequencing was 99.4% with a genome coverage of 18.87× (Figure S1B). Similarly, using the short-read sequencing technology (Miseq, Illumina), a single contig was obtained from the de novo genome assembly method with a 2336.95× coverage. The genome of the bacteriophage fp01 was obtained in a single contig of 109,515 bp with a 39.0% G+C content (Figure 1). The sequenced length did not agree with the previous description of the phage fp01 gDNA molecular weight of ~43.5 Kb, using phage P22 gDNA as a reference in agarose gel electrophoresis [18]. Perhaps, this could be due to differences in its genome topology that could affect migration patterns in agarose gel electrophoresis [57]. We also observed that the genome of the fp01 phage suggests a linear shape (Figure 1). The presence of terminases suggests that genome linearization might occur during DNA packing into the phage capsid [58,59]. This has been reported in *Escherichia coli* and *Salmonella enterica* phages from the order *Caudoviricetes* [45,60].

### 3.2. Annotation, Genome Mapping and Sequence Analysis

The RAST analysis showed 9 subsystems and 17 RNAs (Figure S2A). The PHAST analysis showed 5 categories related to the tail shaft, terminase, base plate, portal, and coat proteins, respectively (Figure S2C). A total of 158 protein-coding genes were identified, among them 100 genes encode hypothetical proteins, 27 genes are associated with DNA packing and transcription, 17 genes are associated with structural and virion assembly, 9 genes are associated with recombination and DNA cleavage, and 5 genes are associated to cell lysis components described below (Supplementary File S1).

DNA packing and transcription identified genes were 2 primases, 1 helicase and 1 ATP-dependent helicase, 2 DNA polymerase III alpha subunit, 3 putative transcriptional regulators, 3 terminase large subunits, and 3 terminase small subunits (File S1). Interestingly, we also identified a D11 and a D14 protein, both described as essential for viral DNA replication in *Escherichia* phages T5-like. Additionally, we identified several genes that encoded for exonucleases and endonucleases associated with DNA recombination and cleavage such as TraG-like protein, recombination endonuclease subunit D12, single-strand DNA (ssDNA) specific exonuclease, and a flap endonuclease.

Structural proteins were also identified, including a capsid maturation protease, a capsid decoration protein, two head morphogenesis protein, a membrane protein as part of the “head structure”, a major tail protein, two minor tail proteins, a tail length tape-measure protein, one tail assembly protein, two baseplate hub protein, a portal protein and two pore-forming tail tip protein were identified a part of the “tail structure” (File S1).

Lysis-associated genes were identified, including toxins such as an endolysin, a lysozyme, a u-spanin protein, an NrdH family redoxin and two MazF family toxin-antitoxin system.

### 3.3. Comparative Genomics and Phylogenetic Analysis

**Figure 2:**
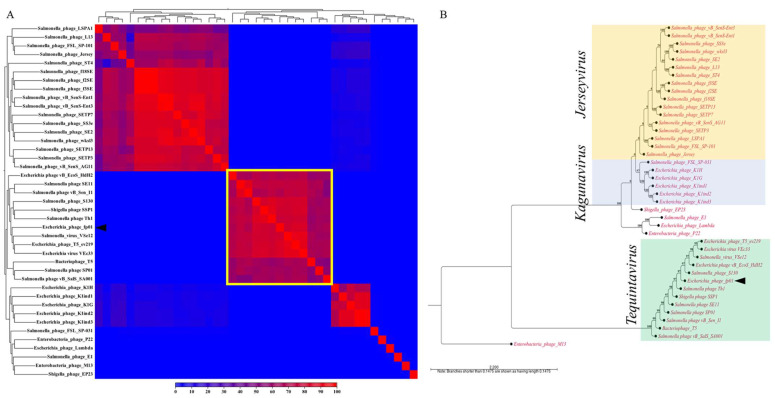
Comparative genomic and evolutionary relationships of *Escherichia* phage FP01. (**A**) Heat map analysis visualization based on aligned *Caudoviricetes* bacteriophages’ whole genomes. The color bar below represents the percentage of identity within strains. (**B**) Phylogenetic tree of evolutionary history computed using the Neighbor-Joining method with a bootstrap test of 1000 replicates. The evolutionary distances were computed using the Jukes-Cantor method. Forty-one complete genomes were used for the genome alignment where Enterobacteria phage M13 was set up as outgroup. Ambiguous positions were removed for each sequence pair (pairwise deletion option). Analyses were conducted using CLCBio (v20.0).

Whole-genome analysis was performed using phages genomes from the *Caudoviricetes* class: *Chaseviridae* (*Myoviridae*), *Autographiviridae* (*Podoviridae*), *Demerecviridae* families, and the *Guernseyvirinae* subfamily. Genera within the *Caudoviricetes* class, are *Jerseyvirus*, *Kagunavirus*, *Lambdavirus*, *Lederbergvirus*, and *Tequintavirus* (*T5likevirus*) genus (Table 1). The heatmap showed that the bacteriophage fp01 belongs to the *Tequintavirus* genus, clustering with *Salmonella* phage VSe12, *Escherichia* phage T5_ev219, and *Escherichia* virus VEc33 (Figure 2A). The average nucleotide identity (ANI) between the phages fp01 and T5viruses VSe12, ev219, and VEc33 was 92.19%, 91.53%, and 91.96%, respectively. The highest alignment percentage (AP) of fp01 observed was 84.75% with phage ev219 (Figure S3). Similar results were observed in the phylogenetic analysis where fp01 cluster within *Tequintavirus* phages, closely related to *Salmonella* phage S130 (Figure 2B). An ANI of 92.80% and AP of 82.47% was observed between fp01 and *Salmonella* phage S130 (Figure S3). These results agree with classification based on the International Committee on Taxonomy of Viruses (ICTV), where phages with dsDNA, non-enveloped capsid, and tailed phages belong to the genus *Tequintavirus*, such as fp01 [18].

**Figure 3:**
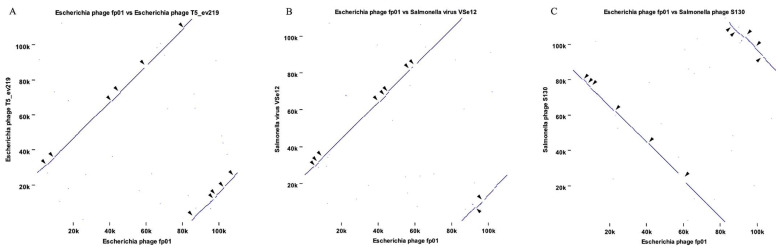
Whole genome comparison by synteny analysis. Genome sequences alignment to identify homology by synteny. (**A**) Comparison between *Escherichia* phage fp01 vs. *Escherichia* phage T5_ev219. (**B**) Comparison between *Escherichia* phage fp01 vs. *Salmonella* phage VSe12. (**C**) Comparison between *Escherichia* phage fp01 vs. *Salmonella* phage S130. Arrows indicate genome gaps within the comparisons. Synteny analysis was also performed for lambda and P22 phages, but homologous regions were not observed. Comparative analysis was computed by using CLCBio (v20.0).

A more detailed analysis of the high genome identity observed between fp01 and T5-such as viruses showed genomes gaps (GGs) and orthologs when comparing phage T5_ev219 (Figure 3A) and phage VSe12 (Figure 3B) to the fp01 genome. Likewise, phage S130 showed a high nucleotide identity to fp01 phage (92.80%) (Figure S3), even though in an inverted orientation when compared with the fp01 genome (Figure 3C). These results indicate that the gene repertory of fp01 is very similar to other lytic members of the *Tequintavirus* genus.

**Figure 4:**
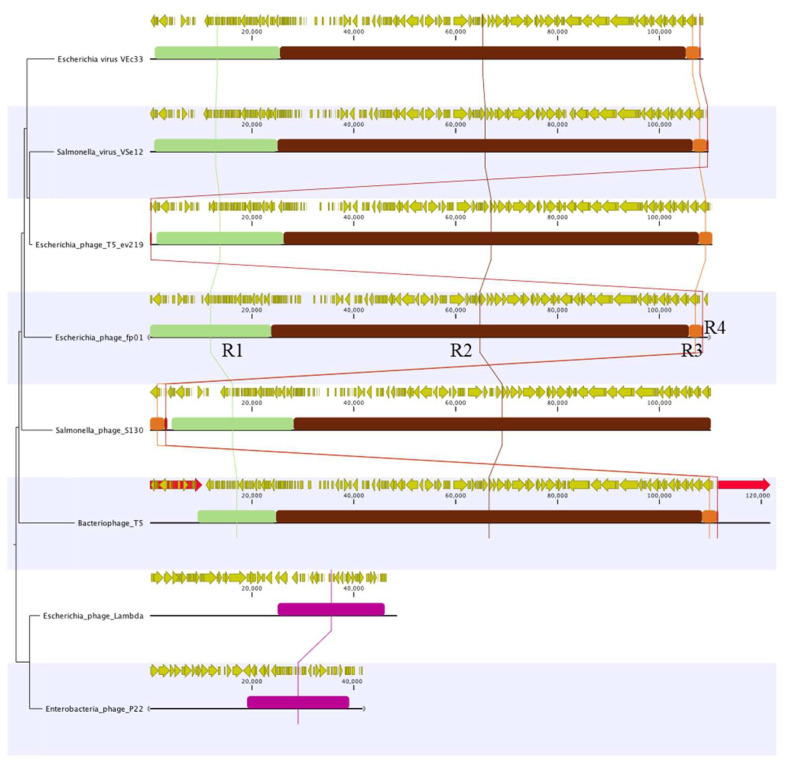
Whole genome analysis of T5-like viruses. Genome sequences were aligned to identify homologous regions by locally colinear blocks. The comparison was performed for Escherichia phage FP01, Salmonella phage VSe12, Escherichia phage T5_ev219, Salmonella phage S130, Escherichia phage VEc33 and Bacteriophage T5. Coding sequences (yellow arrows) and repeat regions (light-red arrows) were included in the analysis. Additionally, Lamba and P22 phages were included in the analysis. However homologous regions with T5viruses were not observed. Comparative analysis was computed by using CLCBio (v20.0).

The comparative analysis of these *Tequintavirus* indicated that their genomes share similar homologous regions, but they have different arrangements. For instance, four locally colinear blocks (LCBs) were identified among the T5-like viruses when compared to the phage fp01 genome (Figure 4). In contrast, no similar LCB was identified when fp01 was compared to a lambda or P22 phages (Figure 4). However, both presented a different LCB located between 20 kb and 40 kb bp of their genomes (Figure 4). Genes associated with replication and structure were identified in LCB 2 (Figure 4, R2-brown LCB), which is the most conserved LCB within these phages. Additionally, genes associated with receptor binding, lysozyme, and lysis were identified in LCB 1 (Figure 4, R1-light green LCB). In contrast, LCB 3 (Figure 4, R3-orange LCB) and LCB 4 (Figure 4, R4-red LCB) presented a small number of coding sequences (CDS) and a single gene that encodes for a hypothetical protein, which seems to be truncated and not conserved among phage genomes (Figure 4).

We also observed that the palindromic repeats were only present in bacteriophage T5 (Figure 4, light-red arrows), indicating that fp01 does not share these regions. However, we believed that fp01 injects or packages its DNA as linear dsDNA due to the presence of terminases, helicases, and primases.

### 3.4. Receptor Binding Interaction Analysis

**Figure 5:**
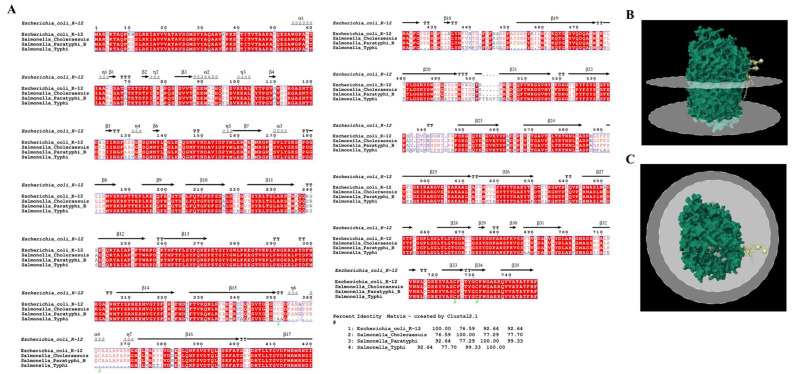
FhuA receptor sequence analysis and protein structure view. (**A**) Amino acid sequences were aligned to identify conserved outer membrane residues among fp01 host strains. The comparison was performed for the FhuA receptor from *E.* *coli* K-12 (reference), *S. enterica* Choleraesuis, Typhi, and Paratyphi B serovars. (**B**,**C**) *E. coli* K-12 FhuA receptor 3D structure was obtained from Protein Data Bank (PDB).

As previously mentioned the bacteriophage fp01 harbor two pore-forming tail-tip, which indicates that fp01 has the ability to interact with a common and conserved liposome-specific receptors, such as FhuA [61], and perhaps gives to the phage fp01 the flexibility to infect *E. coli* and different *S. enterica* serovars. The *fhuA* gene in *E. coli* K-12 has been extensively studied, it encodes for an outer membrane ferric-iron receptor, which additionally serves as a primary receptor for several bacteriophages, including T1, T5, UC-1, and φ80 [22]. *fhuA* gene also is present in *S. enterica* serovars such as Typhi and Paratyphi B, and Choleraesuis. *S. enterica* FhuA has a conserved amino acid sequence and outer membrane regions when compared to *E. coli* K-12 FhuA receptor sequence, with a 76.59%, 92.64%, and 92.64% of identity with Choleraesuis, Paratyphi B, and Typhi serovars, respectively (Figure 5A). These results suggest that conserved binding regions to the FhuA receptor could conferee to fp01 phage its polyvalent characteristic.

We found that the phage fp01 hypothetical protein HWB87_gp108 (YP_009841487.1) (hereafter PBP gp108) has a sequence identity ranging from 66.44 to 98.29% to other PBPs. The lowest identity was observed when compared to *Salmonella* phage SP3, and the highest similarity was observed when compared to *Escherichia* phage vB_EcoS_AKFV33 PBP (Table S1). In contrast, a 95.6% identity was observed between fp01 PBP gp108 and *Escherichia* phage T5_ev219 PBP, as the closest related phage (Figure 2B; Table S1). In the case of *Escherichia* phage T5, the FhuA receptor binds irreversibly with the PBP, gp108 (also called Pb5) [45]. This suggests that the PBP gp108 probably plays a similar role, binding to the FhuA receptor in bacteriophage fp01. Also, we observed a 31% sequence identity within fp01 PBP gp108 and the *Escherichia* phage T5 ATCC 11303-B5 Pb5, the first studied Pb5 PBP [23] (Figure S4; Table S1).

**Figure 6:**
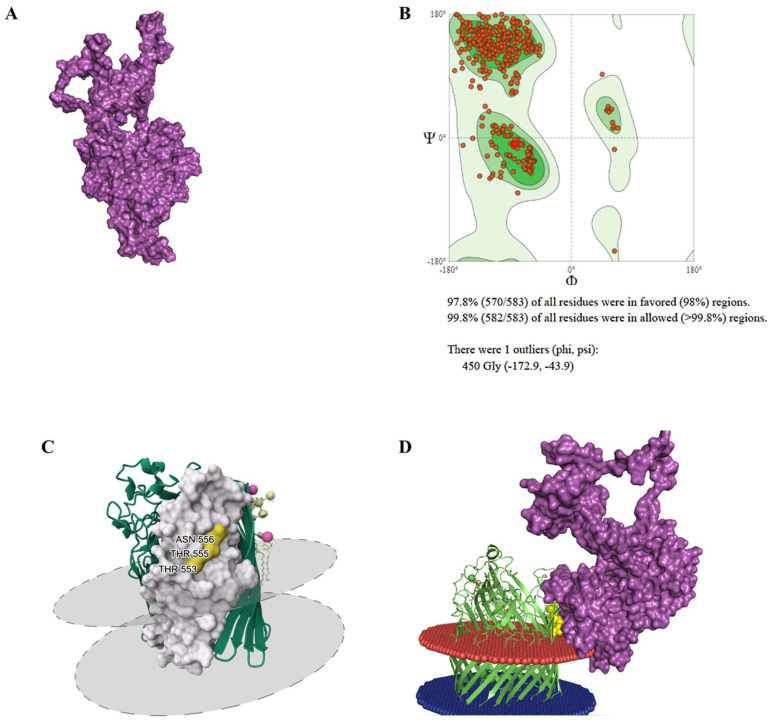
Molecular docking of FhuA protein and hypothetical protein HWB87_gp108. (**A**) 3D model of the FhuA protein produced by PPM 2.0 Web Server using PDB ID: 1FCP. (**B**) Ramachandran plot and statistics. (**C**) 3D hypothetical protein HWB87_gp108 produced by trRosetta program. (**D**) Docked complex of FhuA protein and hypothetical protein HWB87_gp108 created by HDOCK server. The white portion represents the predicted binding site region; the yellow portion and spheres represent the binding amino acid residues in region 552 – 558 (THR553/THR55/TH556) reported to bind with receptor binding protein.

PBP gp108 refined model showed a quality of 97.8% of residues within the Ramachandran favored region after de novo modeling (Figure 6A,B). Binding sites within fp01 PBP gp108 and FhuA receptor were analyzed using in silico molecular docking tools. From the molecular docking analysis, gp108-FhuA interaction was predicted using amino acid residues in the region between PHE485 and LYS611 (Figure S6A). We identified that the binding residues THR553, THR555, and ASN556 were present in the FhuA structure (Figure 6C, yellow portion). Thus, the binding region predicted for the PBP gp108 was between the residues LEU 378 and LEU 568 (Figure 6C, white portion; Figure S5C). Additionally, the predicted interaction between gp108 and FhuA showed a high binding affinity (ΔG) of −10.6 and dissociation constant (K_d_) of 1.8 × 10^−8^ (Figure 6D and Figure S7D). Hence, these results support a possible role of fp01 HWB87_gp108 as PBP, but further analysis is required to confirm this hypothesis.

## 4. Discussion

The wide host range of polyvalent lytic phages made them very attractive for prophylactic control of foodborne bacterial pathogens [13,62,63]. The utilization of bacteriophages as a prophylactic biocontrol has been adopted in the food-producing sectors to reduce the economic burden caused by bacterial infectious diseases [6,63,64]. Currently, phage prophylaxis is applied in different food industries such as dairy [65,66], meat [67,68], and fish [69,70], among others. Bacteriophages can only replicate and multiply through a lytic cycle, where their genetic material does not integrate into the bacterial chromosome and remains as circular plasmids in the cytoplasm, taking over the host machinery for gene transcription, virion assembly (capsid and tails) and DNA packaging, that at the end of their life cycle kill the bacterial cell by endolysin [71,72]. In contrast, when the infecting phage chooses to integrate into the host genome, it enters into a quiescent state becoming a prophage (lysogenic cycle), remaining in that condition indefinitely and being replicated as the host reproduces [24,71]. Usually, phages have a very narrow host range, and bacteriophages that infect multiple species are valuable for fundamental (e.g.; evolution, mechanisms of infection) and practical (e.g.; prophylaxis) studies.

Classification of bacteriophages has been based on their morphology and their type of genetic material, where the main dsDNA families described were denominated Myoviridae (contractile tailed phage), Siphoviridae (long and non-contractile tailed phage), and *Podoviridae* (short-tailed phage); ssDNA families *Microviridae*, and *Inoviridae*; and ssRNA family *Leviviridae* [71,73]. Currently, the taxonomic ranks of *Caudovirales*, *Myoviridiae*, *Siphoviridae* and *Podoviridae* have been abolished by the ICTV and should not be used. The new ICTV classification of phages is based on genomic and proteomic similarities [19,74]. According to the current classification, the bacteriophage fp01 belongs to the class *Caudoviricetes*, family *Demerecviridae*, *Tequintavirus* genus, as a new *Tequintavirus* fp01 species.

The bacteriophage fp01 was isolated using *S. enterica* Choleraesuis VAL201 as the primary host. However, it is able to proliferate in *E. coli* C, *E. coli* B, *E. coli* K12, and *S. enterica* serovars Typhi and Paratyphi B [18]. This indicates that the polyvalent characteristics of the fp01 phage could be related to a common phage receptor among these strains and an RBP, which makes fp01 a potential biocontrol tool for human and animal pathogens in the food-producing sector.

The genome of the fp01 phage was digested with restriction enzymes (*Hind*III and *Hae*III) and estimated to have a genome size close to 42 Kb and similar to P22 and lambda phages [18]. However, fp01 sequenced genome size possess 2.6 times larger genome size than *Salmonella* phage P22 (correct mw = 41,724 bp) [47,75], and 2.2 times larger than lambda phage (48,582 bp) [76]. Additionally, fp01 genome size showed high similarity clustering with *Escherichia* and *Salmonella* phages from the *Tequintaviruses* genera that also belong to the family *Demerecviridae* from the class *Caoudoviricetes* (Figure 2A) [73]. Phylogenetic analysis agreed with the genome heatmap, indicating that fp01 is distantly related to phages lambda and P22 (Figure 2B). In addition, the differences observed within the genome comparison between fp01 and T5, and T5-like viruses (Figure 3) suggest that the phage fp01 could be a variant of a T5-like virus, which agrees with the previous description of polyvalent lytic viruses [77]. Although both the fp01 phage and T5 viruses belong to the *Tequintavirus* genus, they represent different species of phages. It has been reported that T5 group phages were part of the previous *Siphoviridae* family taxonomy based on their *Siphoviridae*-like major tail morphology [78,79]. This indicates that either fp01 or T5 viruses could share a common ancestor besides of the same lytic polyvalent characteristics.

Regarding the fp01 genome annotation, we identified that structural genes correlate with the *Siphoviridae* virion structure of the fp01 phage and its electron microscopy [18]. Interestingly, among the identified replication-associated genes, D11 and a D14, both essential for the early viral replication cycle, have been described in Escherichia phages T5-like and in lambda-like phages [80,81]. This indicates that fp01 uses its DNA-packing machinery for replication.

We did not identify an RNA polymerase, suggesting that fp01 uses bacterial machinery for gene transcription. Additionally, we identified several genes that encoded for exonucleases and endonucleases associated with DNA recombination and cleavage. For instance, TraG-like protein, recombination endonuclease subunit D12, single-strand DNA (ssDNA) specific exonuclease, and a flap endonuclease, which suggest events of recombination during DNA replication [80]. The presence of recombination endonucleases in the genome of fp01 might indicate that this phage recombines with the bacterial chromosome and acquire new properties. For instance, the presence of the *mazF* gene in the fp01 genome suggests that previous recombination events have occurred with an enterobacterial-host chromosome. masF gene has been previously identified in E. coli and described as a lethal toxin that induces a reversible bacteriostasis (cell death) [82,83]. This gene could contribute to fp01 lytic activity and host adaptation, which agrees with fp01 high lytic activity (titers of 5.5 × 10^11^ pfu/mL) and broad host range. The presence of an endolysin, a spanning protein, and a lysozyme agree with fp01 lytic activity as well [84].

The presence of two pore-forming tail tip and a PBP gp108 indicate that fp01 could interact with liposome-specific receptors, such as FhuA. FhuA is a binding receptor for the tail-tip protein pb5 in the bacteriophage T5, which mediates membrane depolarization and phage DNA entrance to the bacterial cytoplasm [61]. The fp01 PBP gp108 sequence showed about 31% of similarity (Figure S4) with the first described Pb5 PBP from phage T5 [23]. However, we identified that fp01 PBP gp108 has a high identity with phage T5_ev219 PBP (Table S1), which indicates that fp01 PBP gp108 might interact with the FhuA receptor. The FhuA protein was reported to bind with phage T5 using amino acid residues 552–558 that are located on the loop 8 [85], whereas PBP Pb5 most likely binds to FhuA protein using amino acids located in positions 89–305 [86]. Here, we identified that the potential binding residues for fp01 PBP in FhuA could be THR553, THR555, and ASN556 (Figure 6A–C), however, we believed that the different binding regions identified in gp108 and Pb5 PBPs could be due to low amino acid sequence identity.

## 5. Conclusions

The polyvalent *Escherichia* phage fp01 has excellent properties for utilization as a prophylactic and therapeutic agent against human and animal bacterial pathogens. Here, we described the genome of the polyvalent phage fp01 and analyzed its phylogenetic relationships based on whole genome analysis. We found that the fp01 phage belongs to the family *Demerecviridae* with a siphovirus morphology, whit a closed relationship with *Escherichia* and *Salmonella* T5 and T5-like phages, that might share a common ancestor with the T5-like siphovirus phages. Additionally, bacteriophage fp01 should be classified as a new *Tequintavirus* fp01 specie according to the current ICTV taxonomy update. The presence of recombination endonucleases such as D11 and D14, in addition to lytic-associated genes such as endolysins, spanning, lysozymes, and *mazF* genes, indicates that fp01 possesses a high lytic activity and are able to acquire genes through its replication that can contribute to its infectivity and host adaptation. Finally, fp01 PBP gp108 showed high identity with several PBPs, especially with the closest related *Escherichia* phage ev219 PBPs, which suggests that gp108 protein might be playing a role in interaction with the common phage receptor FhuA. Perhaps the binding to this common receptor, FhuA by the phage gp108 protein significantly contributes to the polyvalent nature of the fp01 phage. In agreement with our previous observations, a high degree of conservation of the FhuA host cell receptor was observed, which contributes to the ability of these phages to infect multiple genera of Enterobacteriaceae. However, to confirm our insights, further in vitro analyses are required. Its polyvalent characteristic and the high specificity to infect several Enterobacteria make fp01 a promising tool to be used as a food-borne pathogens biocontrol and industrial applications.

## Figures and Tables

**Table 1:** *Caudoviricetes* bacteriophages complete genomes taxonomy list.

Name	Family/Subfamily/ Genus	Accession Number	Reference
*Salmonella* phage SE2	*Jerseyvirus*	JQ007353.1	[37]
*Salmonella* phage ST4		JX233783.1	
*Salmonella* phage vB SenS-Ent2		HG934469.1	[37]
*Salmonella* phage vB SenS-Ent1		HE775250.1	[37]
*Salmonella* phage vB SenS-Ent3		HG934470.1	[37]
*Salmonella* phage SETP3		EF177456.2	[37]
*Salmonella* phage vB SenS AG11		JX297445.1	[37]
*Salmonella* phage SETP13		KF562864.1	[37]
*Salmonella* phage SETP7		KF562865.1	[37]
*Salmonella* phage FSL SP-101		KC139511.1	[37]
*Salmonella* phage LSPA1		KM272358.1	[38]
*Salmonella* phage Jersey		KF148055.1	
*Salmonella* phage SS3e		AY730274.2	[37]
*Salmonella* phage wksl3		JX202565.1	[39]
*Salmonella* phage *f*SE1C		KT962832.1	[40]
*Salmonella* phage *f*SE4C		KT881477.1	[40]
*Salmonella* phage *f*18SE		KR270151.1	[41]
*Salmonella* phage *f*2SE		KU951146.1	Santander Lab
*Salmonella* phage *f*3SE		KU951147.1	Santander Lab
*Escherichia* phage K1G	*Kagunavirus*	GU196277.1	[42]
*Escherichia* phage K1H		GU196278.1	[42]
*Escherichia* phage K1ind1		GU196279.1	[42]
*Escherichia* phage K1ind2		GU196280.1	[42]
*Escherichia* phage K1ind3		GU196281.1	[42]
*Shigella* phage EP23	*Dhillonvirus*	JN984867.1	[43]
*Salmonella* phage STsAS	*Seoulvirus*	MH221128.1	
*Salmonella* phage FSL SP-031	*Guernseyvirinae*	KC139518.1	[37]
*Salmonella* phage E1	*Macdonaldcampvirus*	AM491472.1	[44]
*Salmonella* phage S130	*Demerecviridae*	MH370377.1	[45]
*Salmonella* phage VSe12		NC048794.1	
*Bacteriophage* T5		AY543070	[46]
*Escherichia* phage T5_ev219		LR597655.1	
*Escherichia virus* VEc33		NC_048818	
*Escherichia* phage vB_EcoS_HdH2		NC_048748	
*Salmonella* phage Th1		NC_048795	
*Salmonella* phage SP01		NC_047859	
*Salmonella* phage SE11		NC_048786	
*Salmonella* phage vB_Sen_l1		MT233524	
*Salmonella* phage vB_SalS_SA001		MN445182	
*Enterobacteria* phage P22	*Lederbergvirus*	NC_002371.2	[47]
*Enterobacteria* phage lambda	*Lambdavirus*	J02459.1	[48]
*Enterobacteria* phage M13	*Inoviridae*	NC_003287.2	[49]

Accessed to ICTV taxonomy (21 November 2022).

## Data Availability

Not applicable.

## Supplementary Materials

The following supporting information can be downloaded at: https://www.mdpi.com/article/10.3390/v15020379/s1, File S1: *Escherichia* phage fp01 annotation records; Table S1: Homologous proteins to hypothetical protein HWB87_gp108 identified after a blast search in the NCBI; Figure S1: EPI2ME Basecalling QScore; Figure S2: Distribution of subsystems founds in the polyvalent bacteriophage fp01 genome; Figure S3: Comparative phage fp01 whole genome analysis; Figure S4: Whole genome analysis of T5-like viruses; Figure S5: Tail multiple alignment; Figure S6: Protein-protein complex analysis.
