# Supplementary material for: Comparative Genomics of a Polyvalent Escherichia-Salmonella Phage fp01 and In Silico Analysis of Its Receptor Binding Protein and Conserved Enterobacteriaceae Phage Receptor
Source: Viruses. 2023 Jan 28;15(2):379. doi: 10.3390/v15020379 (PMC9961651; doi:10.3390/v15020379)
Supplement: Supplementary file 1 [file viruses-15-00379-s001.zip › Supplementary figures-Jan12th.pptx]

## Slide 1
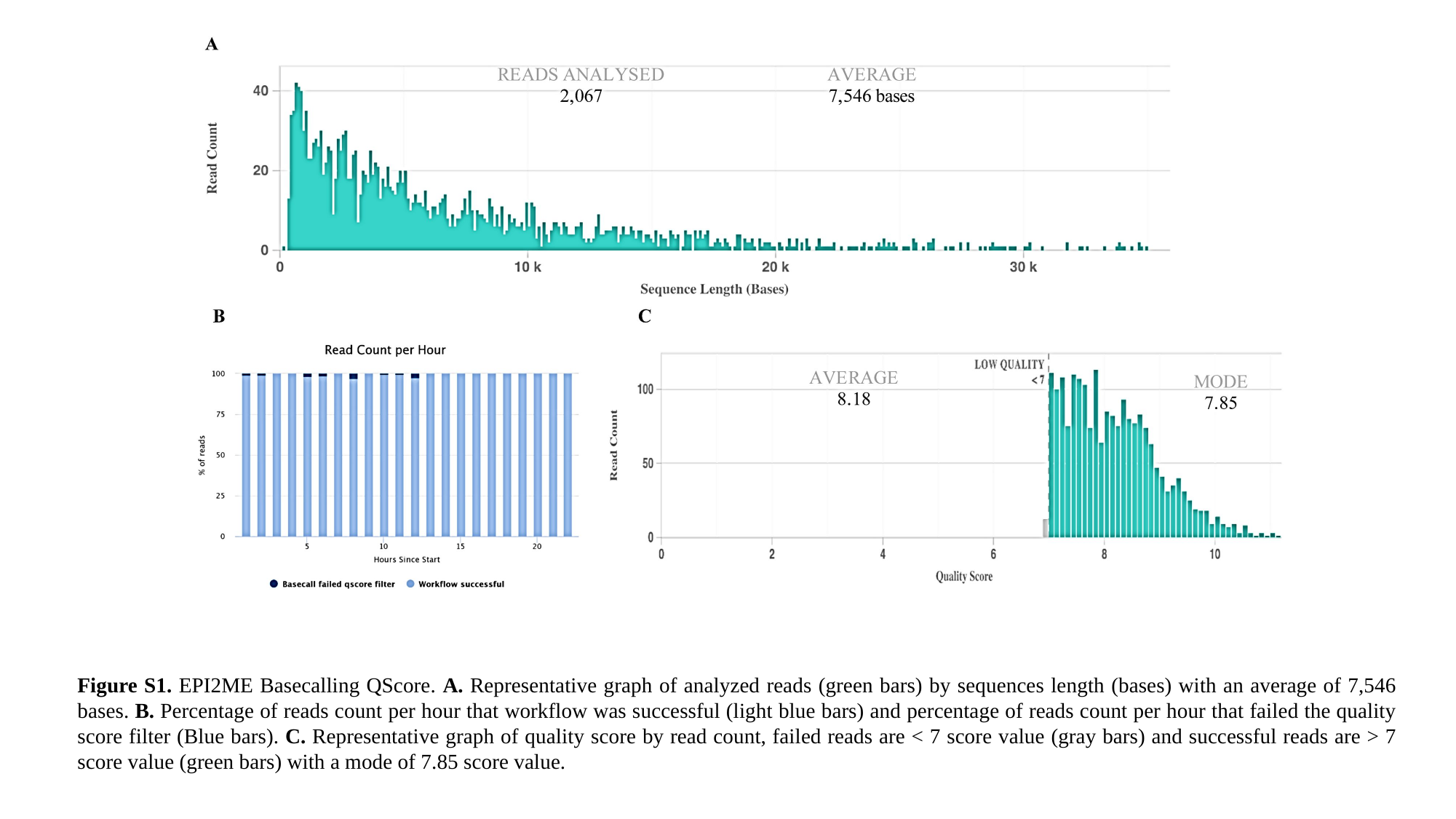

Figure S1. EPI2ME Basecalling QScore. A. Representative graph of analyzed reads (green bars) by sequences length (bases) with an average of 7,546 bases. B. Percentage of reads count per hour that workflow was successful (light blue bars) and percentage of reads count per hour that failed the quality score filter (Blue bars). C. Representative graph of quality score by read count, failed reads are < 7 score value (gray bars) and successful reads are > 7 score value (green bars) with a mode of 7.85 score value.

## Slide 2
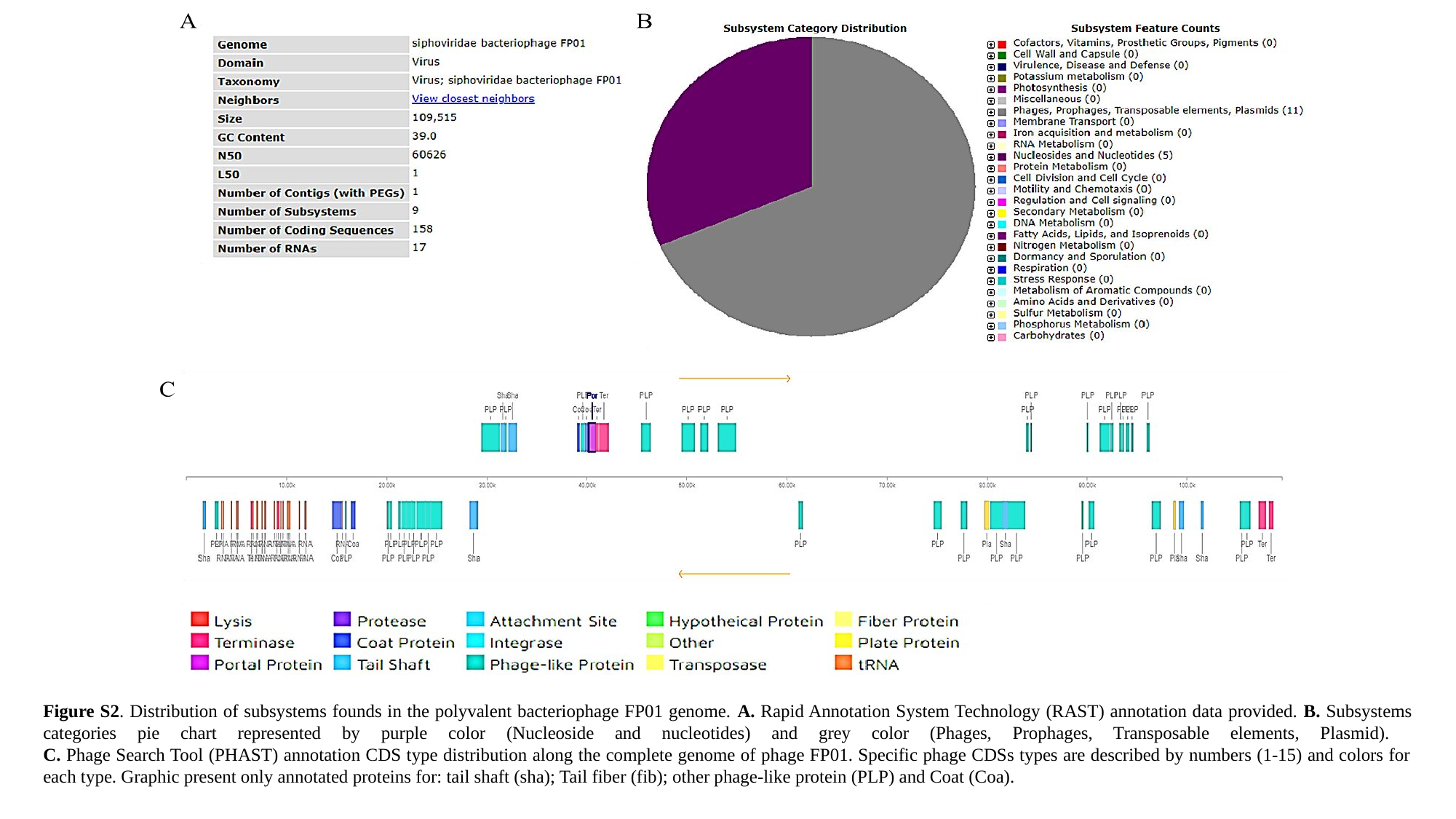

Figure S2. Distribution of subsystems founds in the polyvalent bacteriophage FP01 genome. A. Rapid Annotation System Technology (RAST) annotation data provided. B. Subsystems categories pie chart represented by purple color (Nucleoside and nucleotides) and grey color (Phages, Prophages, Transposable elements, Plasmid).
C. Phage Search Tool (PHAST) annotation CDS type distribution along the complete genome of phage FP01. Specific phage CDSs types are described by numbers (1-15) and colors for each type. Graphic present only annotated proteins for: tail shaft (sha); Tail fiber (fib); other phage-like protein (PLP) and Coat (Coa).

## Slide 3
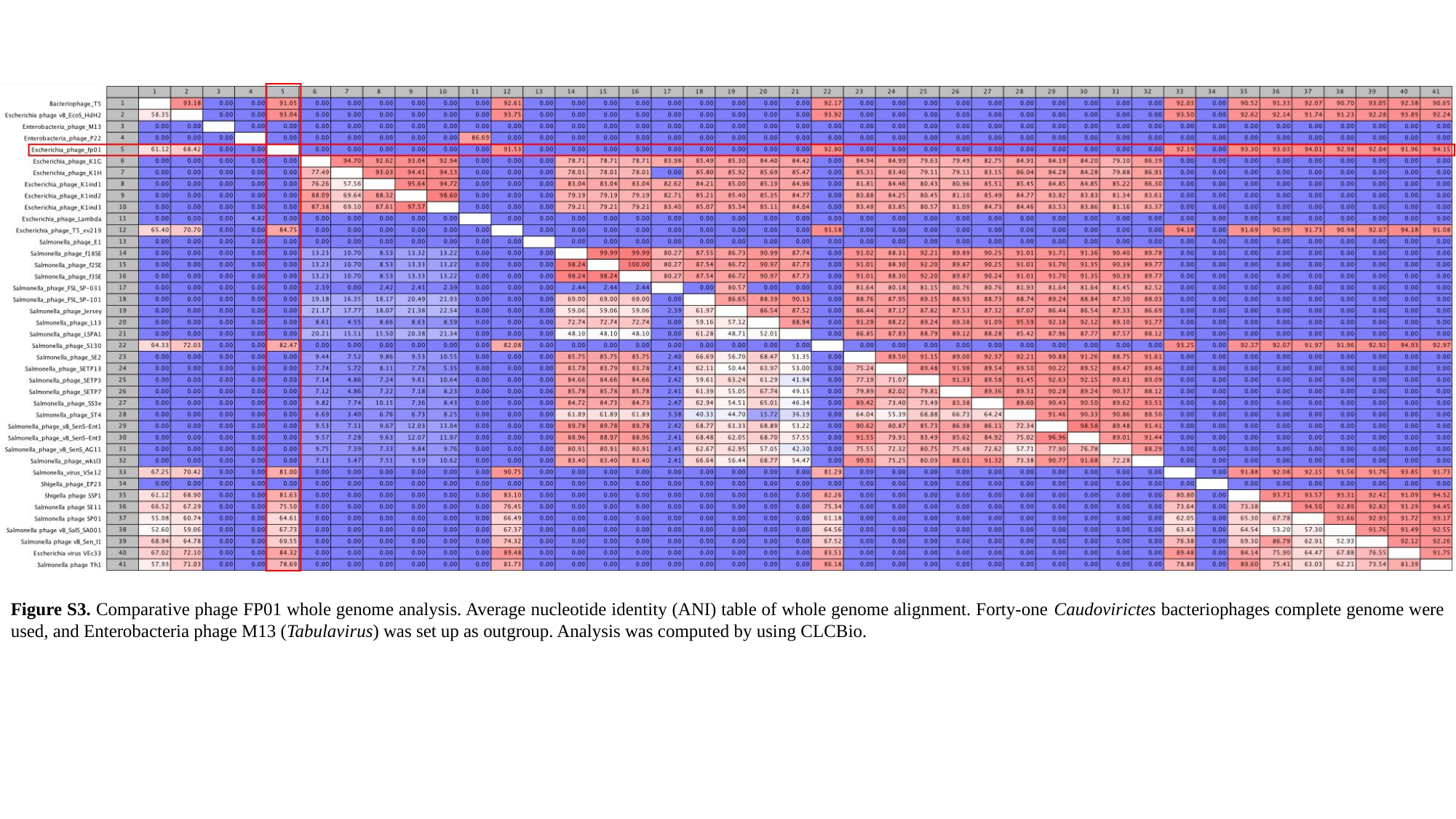

Figure S3. Comparative phage FP01 whole genome analysis. Average nucleotide identity (ANI) table of whole genome alignment. Forty-one Caudovirictes bacteriophages complete genome were used, and Enterobacteria phage M13 (Tabulavirus) was set up as outgroup. Analysis was computed by using CLCBio.

## Slide 4
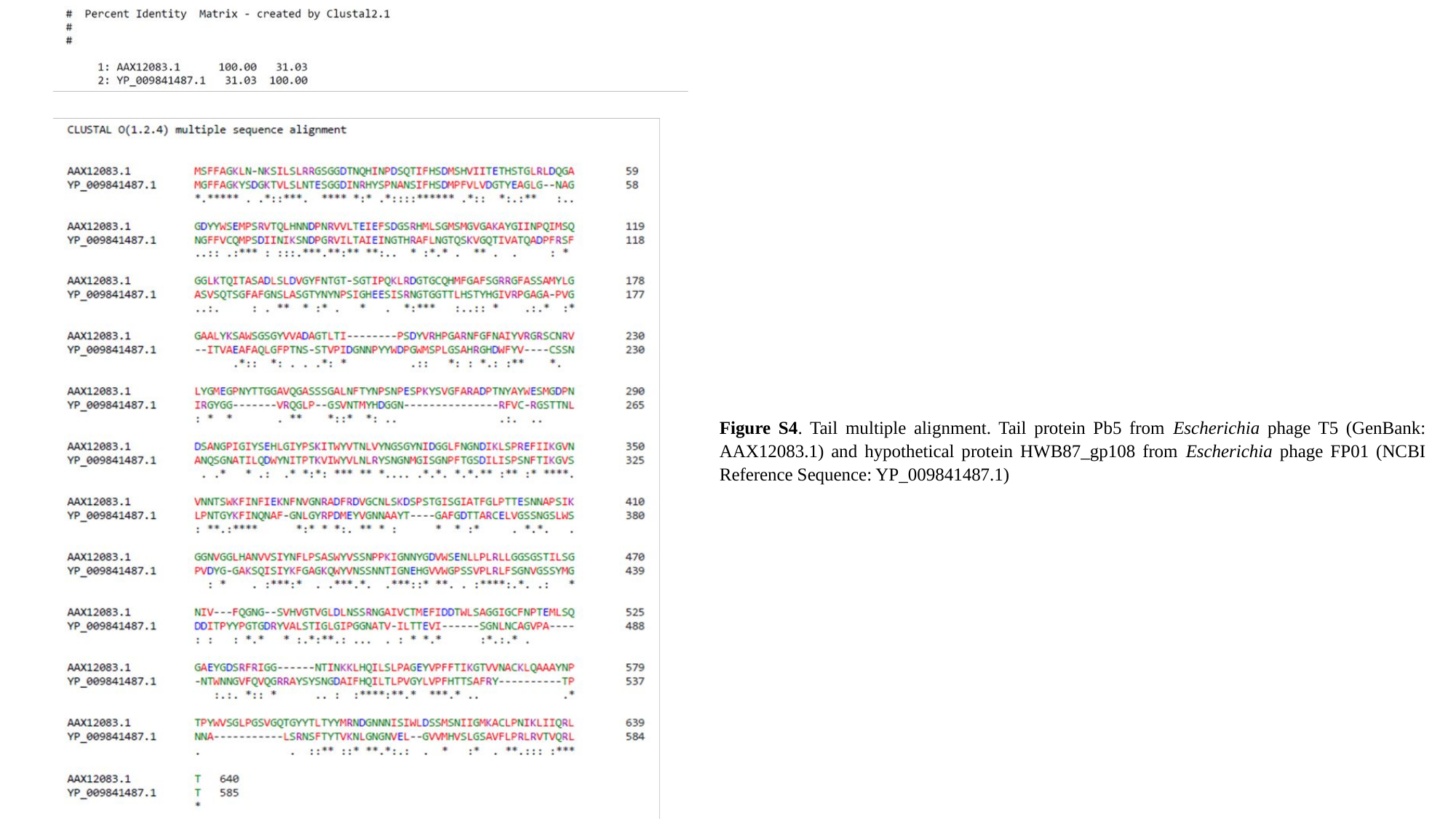

Figure S4. Tail multiple alignment. Tail protein Pb5 from Escherichia phage T5 (GenBank: AAX12083.1) and hypothetical protein HWB87_gp108 from Escherichia phage FP01 (NCBI Reference Sequence: YP_009841487.1)

## Slide 5
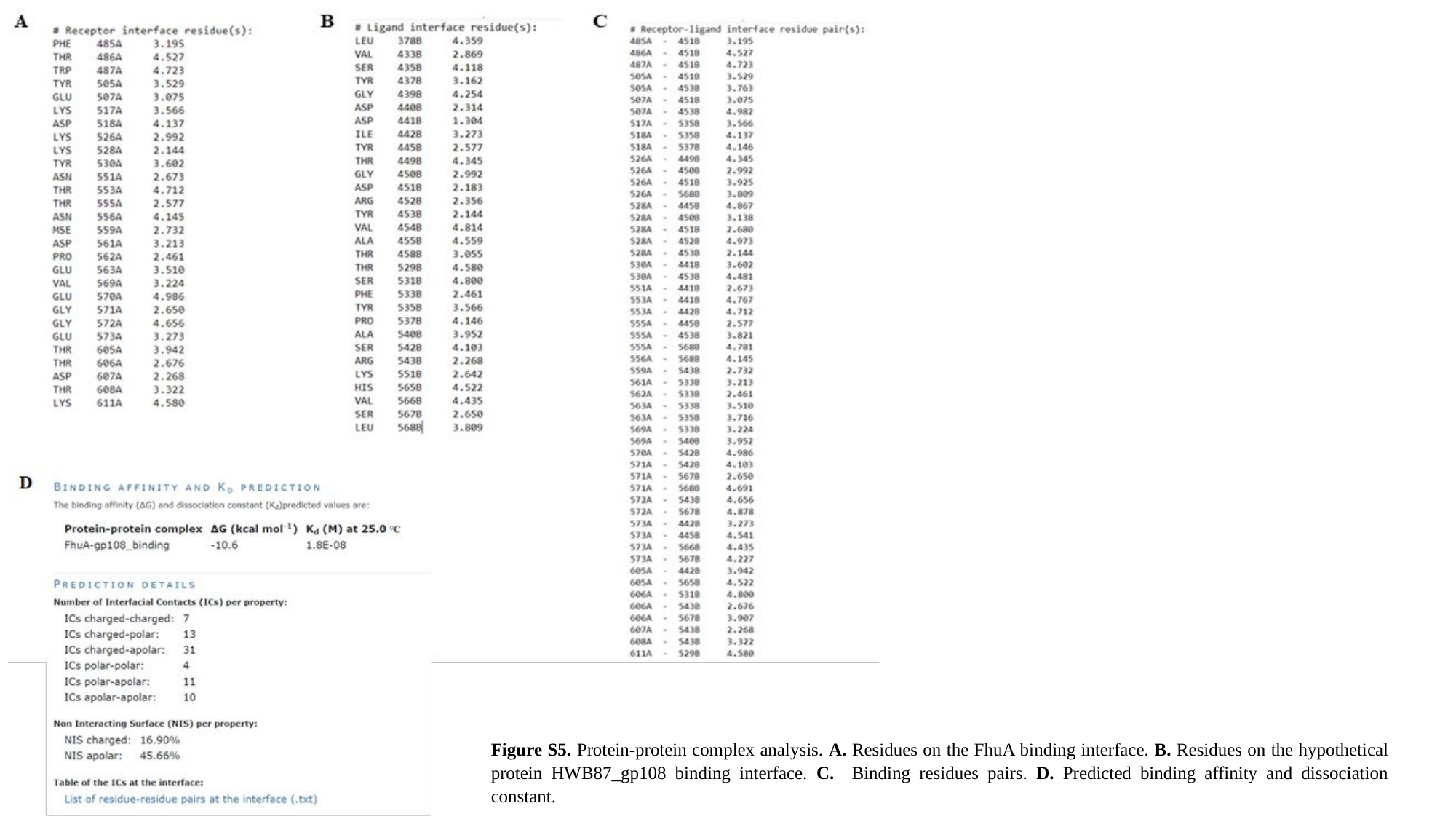

Figure S5. Protein-protein complex analysis. A. Residues on the FhuA binding interface. B. Residues on the hypothetical protein HWB87_gp108 binding interface. C. Binding residues pairs. D. Predicted binding affinity and dissociation constant.
