# Supplementary material for: Comparative Genomics of a Polyvalent Escherichia-Salmonella Phage fp01 and In Silico Analysis of Its Receptor Binding Protein and Conserved Enterobacteriaceae Phage Receptor
Source: Viruses. 2023 Jan 28;15(2):379. doi: 10.3390/v15020379 (PMC9961651; doi:10.3390/v15020379)
Supplement: Supplementary file 1 [file viruses-15-00379-s001.zip › Table_S1.pdf]

Table S1. Homologous proteins to hypothetical protein HWB87_gp108 identified after a blast search in the NCBI.

Description	Scientific Name	Max Score	Total Score	Query Cover	E value	Per. ident	Acc. Len	Accession
hypothetical protein HWB87_gp108 [*Escherichia* phage fp01]	*Escherichia* phage fp01	1183	1183	100%	0	100	585	YP_009841487.1
receptor-binding tail protein [*Escherichia* phage vB_EcoS_AKFV33]	*Escherichia* phage vB_EcoS_AKFV33	1165	1165	100%	0	98.29	585	YP_006382457.1
receptor-binding protein [*Escherichia* phage Lindwurm]	*Escherichia* phage Lindwurm	1165	1165	100%	0	98.29	585	UCR81047.1
receptor-binding tail protein [*Salmonella* phage L6jm]	*Salmonella* phage L6jm	1163	1163	100%	0	98.12	585	YP_009856550.1
receptor-binding tail protein [*Shigella* phage SSP1]	*Shigella* phage SSP1	1159	1159	100%	0	97.61	585	YP_009794581.1
hypothetical protein [*Shigella sonnei*]	*Shigella* sonnei	1158	1158	100%	0	97.61	585	EHE3968306.1
receptor-binding protein [*Salmonella* phage vB_SalS_SA001]	*Salmonella* phage vB_SalS_SA001	1156	1156	100%	0	97.09	585	QKE54301.1
hypothetical protein [*Salmonella enterica* subsp. *enterica* serovar Bareilly]	*Salmonella enterica* subsp. *enterica* serovar Bareilly	1155	1155	100%	0	97.26	585	EBS4928543.1
receptor binding protein [*Escherichia* phage T5_ev212]	*Escherichia* phage T5_ev212	1155	1155	100%	0	97.26	585	VUF55714.1
hypothetical protein [*Escherichia* phage IME178]	*Escherichia* phage IME178	1153	1153	100%	0	96.92	585	QYC97283.1
receptor-binding protein [*Salmonella* phage SP01]	*Salmonella* phage SP01	1150	1150	100%	0	96.92	585	YP_009792475.1
hypothetical protein [*Salmonella enterica* subsp. *enterica* serovar Thompson]	*Salmonella enterica* subsp. *enterica* serovar Thompson	1150	1150	100%	0	96.75	585	EBX4970993.1
receptor-binding protein [*Salmonella* phage LVR16A]	*Salmonella* phage LVR16A	1149	1149	100%	0	96.58	585	YP_009804278.1
putative receptor binding tail protein [*Escherichia* phage JLBYU43]	*Escherichia* phage JLBYU43	1149	1149	100%	0	96.75	585	UGO55636.1
receptor-binding tail tip protein [*Escherichia* phage IrisVonRoten]	*Escherichia* phage IrisVonRoten	1147	1147	100%	0	96.24	585	QXV80353.1
superinfection exclusion protein [*Salmonella* phage 8sent1748]	*Salmonella* phage 8sent1748	1147	1147	100%	0	96.07	585	QNI21575.1
super-infection exclusion protein [*Escherichia* phage phiLLS]	*Escherichia* phage phiLLS	1144	1144	100%	0	95.9	585	YP_009790180.1
receptor-binding tail tip protein [*Escherichia* phage DaisyDussoix]	*Escherichia* phage DaisyDussoix	1144	1144	100%	0	96.41	585	QXV77375.1

putative receptor binding protein [*Escherichia* phage JLBYU40]	*Escherichia* phage JLBYU40	1142	1142	100%	0	95.56	585	UGO55977.1
receptor-binding tail protein [*Salmonella* virus VSe12]	*Salmonella* virus VSe12	1140	1140	100%	0	95.38	585	YP_009849685.1
receptor-binding tail protein [*Escherichia* virus VEc33]	*Escherichia* virus VEc33	1139	1139	100%	0	95.21	585	YP_009853082.1
receptor-binding tail protein [*Escherichia* phage vB_Eco_mar004NP2]	*Escherichia* phage vB_Eco_mar004NP2	1138	1138	100%	0	95.21	585	YP_009824651.1
hypothetical protein HWD11_gp078 [Phage NBEco002]	Phage NBEco002	1138	1138	100%	0	95.38	585	YP_009856968.1
receptor-binding protein [*Escherichia* phage chee24]	*Escherichia* phage chee24	1137	1137	100%	0	95.04	585	YP_009795148.1
receptor binding protein [*Escherichia* phage T5_ev219]	*Escherichia* phage T5_ev219	1135	1135	100%	0	95.04	585	VUF55778.1
receptor-binding tail protein [*Escherichia* phage phiAPCEc03]	*Escherichia* phage phiAPCEc03	1135	1135	100%	0	95.04	585	YP_009785263.1
receptor-binding protein [*Shigella* phage SHSML-45]	*Shigella* phage SHSML-45	1131	1131	100%	0	94.53	585	YP_009280207.1
receptor-binding protein [*Salmonella* phage GEC_vB_N3]	*Salmonella* phage GEC_vB_N3	1131	1131	100%	0	94.7	585	QPI15010.1
hypothetical protein [*Salmonella enterica* subsp. *enterica* serovar Virchow]	*Salmonella enterica* subsp. *enterica* serovar Virchow	1079	1079	94%	0	95.65	553	EBS3195687.1
hypothetical protein VAH1_00171 [*Escherichia* phage vB_EcoS_VAH1]	*Escherichia* phage vB_EcoS_VAH1	984	984	86%	0	95.24	509	QBQ80778.1
receptor binding protein [*Escherichia* virus DT57C]	*Escherichia* virus DT57C	903	903	100%	0	73.04	586	YP_009149909.1
receptor binding protein [*Salmonella* phage vB_StyS-LmqsSP1]	*Salmonella* phage vB_StyS-LmqsSP1	901	901	100%	0	72.87	586	QQM13691.1
hypothetical protein HASG4_00180 [*Escherichia* phage vB_EcoS_HASG4]	*Escherichia* phage vB_EcoS_HASG4	900	900	100%	0	73.21	586	QBQ81433.1
receptor recognition protein [Enterobacteria phage D1G]	Enterobacteria phage D1G	888	888	98%	0	72.54	580	ACN72594.1
receptor-binding protein [*Salmonella* phage vB_SalS_ABTNLsp4]	*Salmonella* phage vB_SalS_ABTNLsp4	870	870	100%	0	72.99	595	QPI13164.1
receptor-binding protein [*Salmonella* phage vB_SenS_S124]	*Salmonella* phage vB_SenS_S124	866	866	100%	0	72.65	595	UDL14130.1
receptor-binding protein [*Salmonella* virus SPC35]	*Salmonella* virus SPC35	863	863	100%	0	72.65	595	YP_004306626.1
hypothetical protein [*Salmonella* phage vB_SalS_ABTNLsp9]	*Salmonella* phage vB_SalS_ABTNLsp9	863	863	100%	0	72.82	595	QPI13621.1
hypothetical protein [*Salmonella enterica* subsp. *enterica*]	*Salmonella enterica* subsp. *enterica*	862	862	100%	0	72.65	595	EDT3055638.1

receptor-binding protein [*Salmonella* phage Th1]	*Salmonella* phage Th1	862	862	100%	0	72.48	595	YP_009849830.1
hypothetical protein HWC04_gp081 [*Escherichia* phage vB_EcoS_HdH2]	*Escherichia* phage vB_EcoS_HdH2	860	860	100%	0	72.48	595	YP_009843474.1
receptor-binding protein [*Salmonella* phage vB_Sen-E22]	*Salmonella* phage vB_Sen-E22	860	860	100%	0	72.82	595	QJD49399.1
hypothetical protein HWD12_gp022 [Phage NBSal003]	Phage NBSal003	859	859	100%	0	72.65	595	YP_009857011.1
receptor binding protein [*Salmonella* phage NR01]	*Salmonella* phage NR01	858	858	100%	0	72.32	595	YP_009283429.1
receptor-binding protein [*Escherichia* phage OSYSP]	*Escherichia* phage OSYSP	853	853	100%	0	72.32	595	YP_009791022.1
hypothetical protein [*Salmonella enterica* subsp. *enterica* serovar Derby]	*Salmonella enterica* subsp. *enterica* serovar Derby	853	853	100%	0	71.14	595	ECJ4201731.1
hypothetical protein [*Salmonella* phage vB_SenS_SB10]	*Salmonella* phage vB_SenS_SB10	839	839	100%	0	69.75	593	QFG07534.1
receptor-binding tail protein [*Salmonella* phage fuchur]	*Salmonella* phage fuchur	838	838	100%	0	69.75	593	YP_009858397.1
receptor-binding protein [*Salmonella* phage S116]	*Salmonella* phage S116	838	838	100%	0	69.58	593	YP_009805278.1
receptor binding protein [*Salmonella* phage BSP22A]	*Salmonella* phage BSP22A	837	837	100%	0	69.41	593	ARM69832.1
receptor-binding protein [*Salmonella* phage 1-23]	*Salmonella* phage 1-23	835	835	100%	0	69.58	593	YP_009819432.1
receptor-binding tail protein [*Salmonella* phage bux]	*Salmonella* phage bux	835	835	100%	0	69.41	593	QIQ61537.1
receptor-binding protein [*Salmonella* phage SE24]	*Salmonella* phage SE24	835	835	100%	0	69.75	593	YP_009848613.1
receptor-binding tail protein [*Salmonella* phage ende]	*Salmonella* phage ende	834	834	100%	0	69.37	590	QIN99960.1
receptor binding protein [Phage vB_SabS_Sds2]	Phage vB_SabS_Sds2	833	833	100%	0	69.41	593	QRV67772.1
receptor-binding tail protein [*Salmonella* phage vaffelhjerte]	*Salmonella* phage vaffelhjerte	833	833	100%	0	69.58	593	QIN99637.1
receptor-binding tail tip protein [*Escherichia* phage TrudiGerster]	*Escherichia* phage TrudiGerster	833	833	100%	0	69.37	590	QXV85240.1
hypothetical protein [*Salmonella enterica*]	*Salmonella enterica*	832	832	100%	0	69.24	593	ECJ3451677.1
receptor-binding tail tip protein [*Escherichia* phage TrudiRoth]	*Escherichia* phage TrudiRoth	832	832	100%	0	69.41	593	QXV85422.1
receptor-binding tail protein [*Salmonella* phage faergetype]	*Salmonella* phage faergetype	832	832	100%	0	69.24	593	YP_009858071.1

receptor-binding protein [*Escherichia* phage vB_EcoS-26175I]	*Escherichia* phage vB_EcoS-26175I	832	832	100%	0	69.04	590	QDJ99943.1
receptor binding protein [*Salmonella* phage S147]	*Salmonella* phage S147	831	831	100%	0	69.24	593	YP_009806052.1
hypothetical protein HWC64_gp078 [*Salmonella* phage OSY-STA]	*Salmonella* phage OSY-STA	831	831	100%	0	69.24	593	YP_009851883.1
receptor-binding protein [*Escherichia* phage saus132]	*Escherichia* phage saus132	831	831	100%	0	69.58	593	YP_009794986.1
receptor-binding tail protein [*Salmonella* phage rokbiter]	*Salmonella* phage rokbiter	830	830	100%	0	69.24	593	YP_009858234.1
receptor-binding tail protein [*Salmonella* phage smaug]	*Salmonella* phage smaug	829	829	100%	0	68.87	590	QIO00945.1
receptor-binding protein [*Salmonella* phage vB_SenS_SB9]	*Salmonella* phage vB_SenS_SB9	829	829	100%	0	69.41	593	QDH47394.1
receptor-binding tail protein [*Salmonella* phage bombadil]	*Salmonella* phage bombadil	828	828	100%	0	69.41	593	YP_009857907.1
receptor-binding tail protein [*Salmonella* phage Seabear]	*Salmonella* phage Seabear	828	828	100%	0	69.08	593	QCQ65488.1
hypothetical protein HOT53_gp024 [*Salmonella* phage SH9]	*Salmonella* phage SH9	828	828	100%	0	69.08	593	YP_009804131.1
receptor-binding protein [*Salmonella* virus Stitch]	*Salmonella* virus Stitch	828	828	100%	0	69.08	593	YP_009146101.1
receptor-binding protein [*Salmonella* phage Seafire]	*Salmonella* phage Seafire	828	828	100%	0	69.41	593	YP_009816816.1
receptor-binding protein [*Salmonella* phage 2-3]	*Salmonella* phage 2-3	828	828	100%	0	69.24	593	YP_009852070.1
receptor-binding tail tip protein [*Escherichia* phage SelmaRatti]	*Escherichia* phage SelmaRatti	828	828	100%	0	69.97	595	QXV84460.1
FhuA receptor-binding tail protein [*Salmonella* phage Sepoy]	*Salmonella* phage Sepoy	828	828	100%	0	69.08	593	YP_009845375.1
receptor-blocking protein [*Salmonella* phage SE3]	*Salmonella* phage SE3	827	827	100%	0	69.24	593	QEG07468.1
receptor-binding tail protein [*Salmonella* phage rutana]	*Salmonella* phage rutana	827	827	100%	0	69.08	593	QIO02001.1
receptor-binding tail protein [*Salmonella* phage vB_SenS-3]	*Salmonella* phage vB_SenS-3	827	827	100%	0	68.87	590	QIN93530.1
hypothetical protein [*Salmonella* phage 8sent65]	*Salmonella* phage 8sent65	827	827	100%	0	69.24	593	QNI21950.1
receptor-binding tail protein [*Salmonella* phage polluks]	*Salmonella* phage polluks	827	827	100%	0	68.87	590	QIO00289.1
hypothetical protein [*Salmonella enterica* subsp. *enterica* serovar Typhimurium]	*Salmonella enterica* subsp. *enterica* serovar Typhimurium	827	827	100%	0	68.91	593	EDV2866874.1

receptor-binding protein [*Escherichia* phage vB_Eco_mar003J3]	*Escherichia* phage vB_Eco_mar003J3	826	826	100%	0	69.24	593	YP_009824387.1
hypothetical protein [*Salmonella enterica* subsp. *enterica*]	*Salmonella enterica* subsp. *enterica*	822	822	100%	0	69.58	593	EDY0344208.1
hypothetical protein HWC37_gp093 [*Salmonella* phage vB_SenS_SB13]	*Salmonella* phage vB_SenS_SB13	821	821	100%	0	69.58	593	YP_009848214.1
receptor-binding protein [*Salmonella* phage STG2]	*Salmonella* phage STG2	820	820	100%	0	69.41	593	YP_009815133.1
receptor-binding protein [*Salmonella* phage JNwz02]	*Salmonella* phage JNwz02	820	820	100%	0	69.58	593	QYC50589.1
receptor-binding tail tip protein [*Escherichia* phage SuperGirl]	*Escherichia* phage SuperGirl	820	820	100%	0	69.41	593	QXV84736.1
receptor-binding tail tip protein [*Escherichia* phage HildyBeyeler]	*Escherichia* phage HildyBeyeler	820	820	100%	0	67.95	595	QXV80181.1
receptor-binding tail protein [*Salmonella* phage falkor]	*Salmonella* phage falkor	818	818	100%	0	69.24	593	QIO01834.1
receptor-binding tail protein [*Salmonella* phage STWB21]	*Salmonella* phage STWB21	815	815	100%	0	68.18	593	QTJ63420.1
receptor-binding tail protein [*Salmonella* phage Sw2]	*Salmonella* phage Sw2	805	805	100%	0	68.01	593	YP_009812307.1
receptor-binding protein [*Escherichia* coli phage vB_EcoS_Ace]	*Escherichia* coli phage vB_EcoS_Ace	803	803	100%	0	67.85	593	QNR52195.1
receptor-binding tail protein [*Salmonella* phage bastian]	*Salmonella* phage bastian	802	802	100%	0	67.56	593	YP_009858562.1
receptor binding protein [*Salmonella* phage 1-19]	*Salmonella* phage 1-19	800	800	100%	0	67.9	593	YP_009853229.1
receptor-binding tail protein [*Salmonella* phage gmork]	*Salmonella* phage gmork	800	800	100%	0	68.24	593	QIO01163.1
hypothetical protein [*Salmonella enterica* subsp. *enterica* serovar Hadar]	*Salmonella enterica* subsp. *enterica* serovar Hadar	798	798	100%	0	68.4	593	EDV1300518.1
receptor binding protein [*Salmonella* phage S132]	*Salmonella* phage S132	796	796	100%	0	67.73	593	YP_009805728.1
receptor-binding protein [*Salmonella* phage 100268_sal2]	*Salmonella* phage 100268_sal2	795	795	100%	0	67.73	593	YP_009320892.1
receptor-binding tail protein [*Salmonella* phage bobsandoy]	*Salmonella* phage bobsandoy	795	795	100%	0	67.73	593	QIO01330.1
receptor-blocking protein [*Salmonella* phage SP3]	*Salmonella* phage SP3	795	795	100%	0	66.44	595	YP_009804586.1
